# Factors Affecting Distribution of Pharmaceutically Active Compounds in Bottom Sediments of Odra River Estuary (SW Baltic Sea)

**DOI:** 10.3390/molecules30193935

**Published:** 2025-10-01

**Authors:** Joanna Giebułtowicz, Dawid Kucharski, Grzegorz Nałęcz-Jawecki, Artur Skowronek, Agnieszka Strzelecka, Łukasz Maciąg, Przemysław Drzewicz

**Affiliations:** 1 Department of Drug Chemistry, Pharmaceutical and Biomedical Analysis, Faculty of Pharmacy, Medical University of Warsaw, Banacha 1, 02-097 Warszawa, Poland; 2 Department of Toxicology and Food Sciences, Faculty of Pharmacy, Medical University of Warsaw, Banacha 1, 02-097 Warszawa, Poland; 3 Institute of Marine and Environmental Sciences, University of Szczecin, Mickiewicza 16, 70-383 Szczecin, Poland; 4 Cement Roadstone Holdings Central Lab, Karsy 77, 27-530 Ożarów, Poland; 5 Polish Geological Institute-National Research Institute, Rakowiecka 4, 00-975 Warszawa, Poland

**Keywords:** pharmaceutically active compounds, bottom sediments, Odra River estuary, environmental pollution, spatial distribution, multivariate statistical analysis

## Abstract

The results from previous environmental studies on the physicochemical properties of bottom sediments from the Odra River estuary (SW Baltic Sea) and their contamination by pharmaceutically active compounds (PhACs) were compiled and analyzed by the use of various statistical methods (Principal Component Analysis, ANOVA/Kruskal–Wallis, Spearman correlation analysis, Partial Least Squares Discriminant Analysis, and Cluster Analysis). These studies included data on 130 PhACs determined in sediment samples collected from 70 sites across the Odra River estuary as well as the site distance to wastewater treatment plant discharge, PhACs’ physicochemical properties (Kd, Kow, pKa, solubility, metabolism), and sales data. Additionally, total organic carbon, total nitrogen, total phosphorus, acid volatile sulfides, clay mineral content, and trace elements such as As, Ba, Cd, Co, Cr, Cu, Fe, Hg, Mn, Mo, Ni, Pb, Sn, and Zn were analyzed. Clay mineral content and TP were identified as the key physicochemical factors influencing the spatial distribution of PhACs in bottom sediments, exerting a greater impact than the distance of sampling sites from WWTP discharge points. The distribution of PhACs in the estuary was also influenced by the Kd and solubility of the compounds. More soluble pharmaceuticals with low adsorption affinity to sediments were detected more frequently and transported to distant locations, whereas less soluble compounds with high adsorption affinity settled down in bottom sediments near contamination sources. Neither the proportion of a drug excreted unchanged, nor its prescription frequency and sales volume, influenced the spatial distribution of PhACs. In general, Kd may be a useful parameter in the planning of environmental monitoring and tracing migration of PhACs in aquatic environments.

## 1. Introduction

Pharmaceutically active compounds (PhACs) are substances used to prevent, diagnose, or treat diseases and regulate organic functions [1]. Currently, approximately 4000 molecules with diverse physicochemical and biological properties, as well as distinct biochemical mechanisms, are part of the drug repertoire [2,3]. The pharmaceutical industry continuously develops PhACs with enhanced resistance to biochemical degradation to improve therapeutic efficacy, reduce required dosages, and minimize the harmful effects of metabolites on human and animal health. Thus, many PhACs are excreted unchanged as parent compounds from the body. When metabolized, they often form conjugates that increase polarity and water solubility while reducing their n-octanol-water partition coefficient (Kow) [4]. Under specific conditions, for example those present in wastewater treatment plant (WWTP) or bottom sediments, conjugation reactions can reverse, regenerating the parent compounds [4,5,6].

Excreted PhACs enter WWTPs mainly with wastewater, where they are removed through adsorption onto activated sludge or biodegradation [7]. Some PhACs used in livestock and aquaculture enter the environment directly [8,9,10]. However, dissociated and conjugated PhACs poorly adsorb to sludge and are not effectively removed. Some PhACs, such as tetracyclines and fluoroquinolones, form water-soluble metal complexes [11,12,13], which may adsorb onto suspended organic particles in WWTP effluents. Moreover, many PhACs degrade slowly in comparison to the biological removal rate, making WWTPs significant sources of PhAC pollution in aquatic environments [5,14,15].

In aquatic environments, PhACs can undergo direct photolysis when absorbing solar radiation, while others degrade via indirect photolysis, reacting with radicals derived from dissolved organic matter, nitrates, and other natural water constituents [16,17]. However, most PhACs accumulate in bottom sediments, where photodegradation occurs very slowly [2]. These compounds can be re-suspended into the water column due to hydrodynamic forces, such as wind-induced flow, floods, ship movement, or waterway maintenance activities, leading to their long-range transport. The adsorption of PhACs in sediments depends on many factors, such as the lipophilicity and dissociation properties of the drugs as well as the pH, salinity, and temperature of the water, and many other factors [10,18,19,20].

Generally, PhACs with log Kow > 5 and high molecular weights readily adsorb to sediments and are removed from the water column [3,7,21,22]. Many PhACs possess dissociable functional groups (acidic or basic). If their acid dissociation constant (pKa) falls within the typical surface water pH range (5–8), their sorption is influenced by mineral composition, particularly clay content in sediments [23,24,25]. In humic acid macromolecules, carboxyl groups dissociate at pH > 4, while phenolic groups dissociate at pH > 8. Increasing pH enhances the negative charge of humic acids by promoting carboxyl deprotonation, leading to charge repulsion that prevents adsorption of some dissociated PhACs on the surface of bottom sediment particles. However, the sediment surface charges are reduced by metal cation adsorption. Such bound metals with residual charge may form complexes with pharmaceuticals and immobilize the compounds in sediments. PhACs may also adsorb as neutral complexes or conjugates through hydrogen bonds or van der Waals interactions. Adsorption behavior of PhACs is also affected by factors such as total organic carbon, salinity, cation exchange capacity, and overall environmental conditions, making predictive modeling difficult [22]. Furthermore, European studies suggest that PhAC occurrence in the environment did not always correlate with consumption levels [26].

Most research focuses on PhACs in surface and groundwater [27,28,29]. Based on these findings, the European Union (EU) has taken regulatory steps to mitigate water contamination [30]. Under Directive 2013/39/EU [31], a Watch List (WL) of emerging pollutants was introduced in order to enhance information on their environmental fate and risk, which was to be used in preventative action against water contamination. The WL is periodically updated based on environmental monitoring results. While PhACs are routinely monitored in natural waters, recent studies highlight their accumulation in bottom sediments, which act as repositories for those pollutants [32,33]. Concentrations of pharmaceuticals in sediments often exceed those in surface waters due to photolysis at the water surface and their deposition with water-suspended material on the riverbed [19,34]. Continuous accumulation of persistent contaminants in sediments may reach concentrations resulting in ecotoxic effects [35,36,37,38]. Since sediments support diverse organisms crucial for aquatic ecosystems [39], PhAC adsorption is one of the critical factors influencing the bioavailability and ecological risks of the compounds [40,41,42].

Most PhAC adsorption studies use batch experiments, which do not reflect real aquatic conditions [43]. In flowing river environments, PhAC adsorption differs from steady-state batch experiments [44]. Consequently, sediment retention of PhACs in natural systems may deviate from laboratory predictions. Additionally, limited adsorption data are known for many PhACs. Aquatic organisms can accumulate PhACs from water, subsequently settling in sediments upon death [45,46,47,48,49]. Other sediment pollutants, such as heavy metals, may enhance PhAC immobilization in the river bottom [50]. Hydrolysis and biodegradation reduce PhAC concentrations in sediments, but these processes are too complex to be replicated experimentally. Thus, understanding PhAC distribution in the environment requires multivariate studies integrating physicochemical properties, anthropogenic influences, and environmental conditions. Altogether, the mobility and fate of the pollutants in the aquatic environment are affected by the complex interaction of compound- and sediment-specific processes, which, in turn, depend on the physicochemical properties of the compound and sediment [51,52,53]. Mathematical modelling of those processes and elucidation of pollutant fate and mobility is very difficult or even impossible due the paucity of data. In most cases, elucidation of the behavior of pollutants in the environment was concluded based on laboratory studies of adsorption on sediments, biodegradation, photolysis, solubility, and so on [54].

In this study, for the first time, real environmental data from previous studies on the Odra River estuary [55,56] were analyzed. The study included data on 130 PhACs determined in sediment samples collected from 70 sites across the Odra River estuary, as well as the site distance to WWTP discharge, PhACs’ physicochemical properties (Kd, Kow, pKa, solubility, metabolism), and sales data. Additionally, total organic carbon (TOC), total nitrogen (TN), total phosphorus (TP), acid volatile sulfides (AVS), clay mineral content, and trace elements such as As, Ba, Cd, Co, Cr, Cu, Fe, Hg, Mn, Mo, Ni, Pb, Sn, and Zn were analyzed. Statistical methods such as Principal Component Analysis, ANOVA/Kruskal–Wallis, Spearman correlation analysis, Partial Least Squares Discriminant Analysis, and Cluster Analysis were applied in the analysis. Results of a multivariate statistical analysis of sediment and PhAC parameters revealed environmental and anthropogenic factors affecting the spatial distribution of PhACs in the riverine system. The identified factors were described and discussed in terms of their environmental relevance. Additionally, a list of PhACs that showed a strong tendency for retention in bottom sediment was proposed.

## 2. Study Area

**Figure 1:**
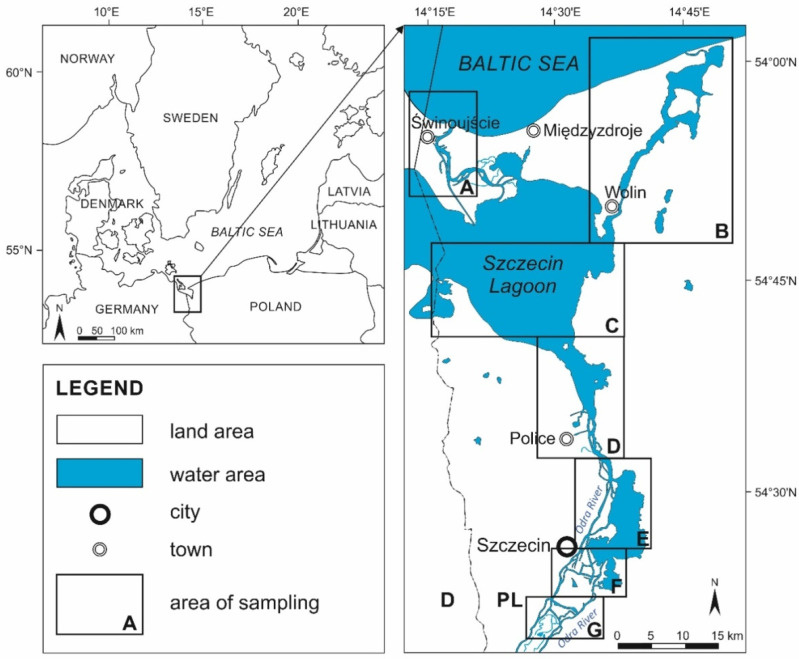
The map of the sampling area. The Odra River estuary includes the following areas: (**A**)—Świna Strait, (**B**)—Dziwna Strait, (**C**)—the middle part of the Szczecin Lagoon, (**D**)—Roztoka Odrzańska, the southern part of the Szczecin Lagoon, (**E**)—the northern part of Lake Dąbie, (**F**)—the port of Szczecin, and the southern part of Lake Dąbie, and (**G**)—the Międzyodrze, a lowland between eastern and western branches of the Odra River.

The Odra River estuary in northwestern Poland features valuable natural habitats alongside highly urbanized and industrialized areas (Figure 1). It includes water bodies such as the Szczecin Lagoon, Western and Eastern branches of the Odra River, Roztoka Odrzańska, Kamieński Lagoon, and Lake Dąbie [57]. The Szczecin Lagoon connects to the Baltic Sea through the Peene (west), Świna, and Dziwna Straits (east).

The northern part of the estuary, designated as a Natura 2000 site, is sparsely populated. The lagoon’s hydrodynamics are driven more by wind-induced seawater intrusion or water circulation than by river flow. Water circulation is more characteristic of a lagoon than an estuary; however, the systematic influence of backwater flows caused by the northwest wind extends several dozen kilometers up the Odra River. Although there are no tides, the results of water movement are very similar. Freshwater from the Odra River mixes with seawater entering during storms and strong winds, affecting salinity, temperature, and contaminant distribution. Water exchange with the Pomeranian Bay depends on meteorological conditions, sea level, and river discharge. About 60–70% of water flows through the Świna Strait, while the Peene and Dziwna each account for 15–20% [58,59]. The lagoon’s average depth is less than 6 m.

The Odra estuary is highly industrialized, hosting shipyards, seaports, and the “Grupa Azoty Police” chemical plant. Szczecin, the largest city in the region, has over 400,000, while Świnoujście has about 40,000 inhabitants. The two major ports in the region are located in these cities. The marine traffic between Szczecin and the Baltic Sea transits through the 67 km long waterway across the lagoon. There are numerous marinas and small ports used for sailing activities in the lagoon area. The estuary is a natural buffer which diminishes the movement of pollutants into the Baltic Sea.

The Odra River, the third-largest in the Baltic region, has a length of 866 km and a catchment area of 122,512 km^2^, located mostly in Poland. Its annual discharge to the Baltic reaches 18.5 billion m^3^. The river, flowing through urbanized and industrial zones, receives wastewater from numerous WWTPs, mines, and factories. Additional inflows of the contaminated water into the Szczecin Lagoon are from rivers: Gowienica (51 km, Poland), Uecker (98 km, Germany), Zarow (16 km, Germany), and Peene (129 km, Germany). The system of drainage ditches in farmland areas is also responsible for PhAC-contaminated water runoff from farmland into those rivers.

The City of Szczecin operates two major WWTPs with advanced nutrient removal: “Pomorzany” (PE = 417,000, Q = 66,000 m^3^/d) and “Zdroje” (PE = 177,000, Q = 18,000 m^3^/d), modernized in 2008–2009 to meet EU standards. Other WWTPs in the estuary region serve towns such as Dziwnów, Wolin, Kamień Pomorski, Stepnica, Goleniów, Gryfino, Police, Kołbaskowo, Nowe Warpno, and Międzyzdroje, collectively treating wastewater from over 95% of the local population. However, leaks and overflows during heavy rainfall may contribute to surface and groundwater contamination by PhACs [60,61].

Agricultural activities pose additional risks, particularly from livestock farms. Manure, often spread untreated on fields, can introduce pharmaceutical contaminants into the environment. While anaerobic fermentation in biogas plants can reduce pharmaceutical content, such facilities are scarce in the area around the lagoon. Even subsurface manure application does not fully prevent contaminant leaching from the farmland.

Deepening and maintenance of navigation channels and port basins by dredging of the bottom sediments also induced mobilization of contaminants and consequently their movement in the environment. Dredged sediments, often dumped in nearshore areas, may release pollutants back into the lagoon. Recently, dredged material was used to create artificial islands in the lagoon as wildlife refuges. However, during weathering of deposited sediments, contaminants may be released into surrounding waters. Detailed descriptions of sampling sites collected from the estuary are given in [55,56].

## 3. Results and Discussion

### 3.1. Influence of Physical and Chemical Characteristics of Sediments on the Occurrence of PhACs

**Figure 2:**
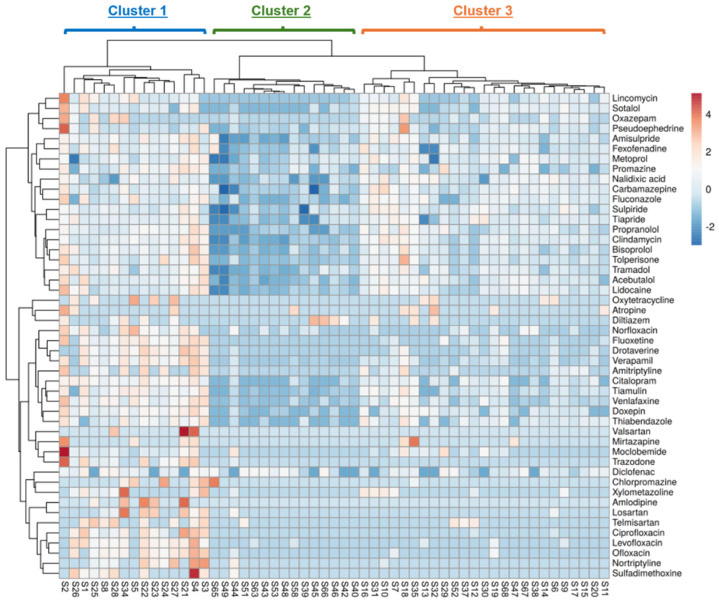
Cluster heatmap presenting interrelations between sediment samples and PhAC composition profile. The colors from blue to red represent the standardized parameters of the bottom sediments from the lowest to the highest values.

Cluster Analysis is a commonly used method to reveal patterns and structures within multivariate environmental datasets that may provide insight into underlying relationships and associations among samples and environmental parameters. A heatmap and a two-dimension dendrogram were used for a graphical overview of the interrelations between sediment samples and their composition. The PhACs present in at least 10% of the sediment samples were taken to statistical analysis. The clustering of sediment samples characterized by similar profiles of PhACs and PhAC clusters with similar trends of content in the samples can be observed in the heatmap (Figure 2). The first cluster from the left comprises the samples with a high content of PhACs, whereas the second one comprises samples with low content, and the third one comprises samples with medium content.

**Figure 3:**
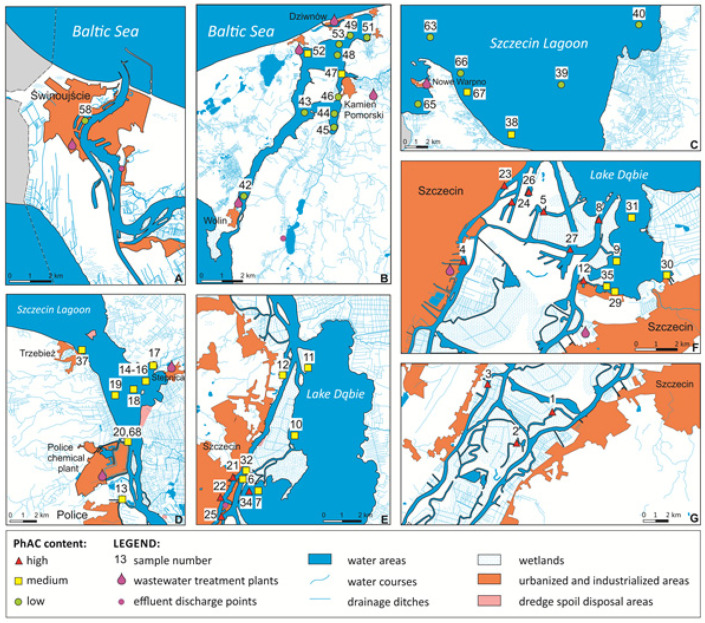
The map of the sampling sites in the Odra River estuary grouped in clusters based on PhAC content. (**A**)—Świna Strait, (**B**)—Dziwna Strait, (**C**)—the middle part of the Szczecin Lagoon, (**D**)—Roztoka Odrzańska, the southern part of the Szczecin Lagoon, (**E**)—the northern part of Lake Dąbie, (**F**)—the port of Szczecin, and the southern part of Lake Dąbie, and (**G**)—the Międzyodrze, a lowland between eastern and western branches of the Odra River.

The samples grouped in clusters with high, medium, and low PhAC content, shown in Figure 2, are marked with red triangles, yellow squares, and green circles, respectively. The location of sampling points in the Odra River estuary is presented on the map (Figure 3).

**Figure 4:**
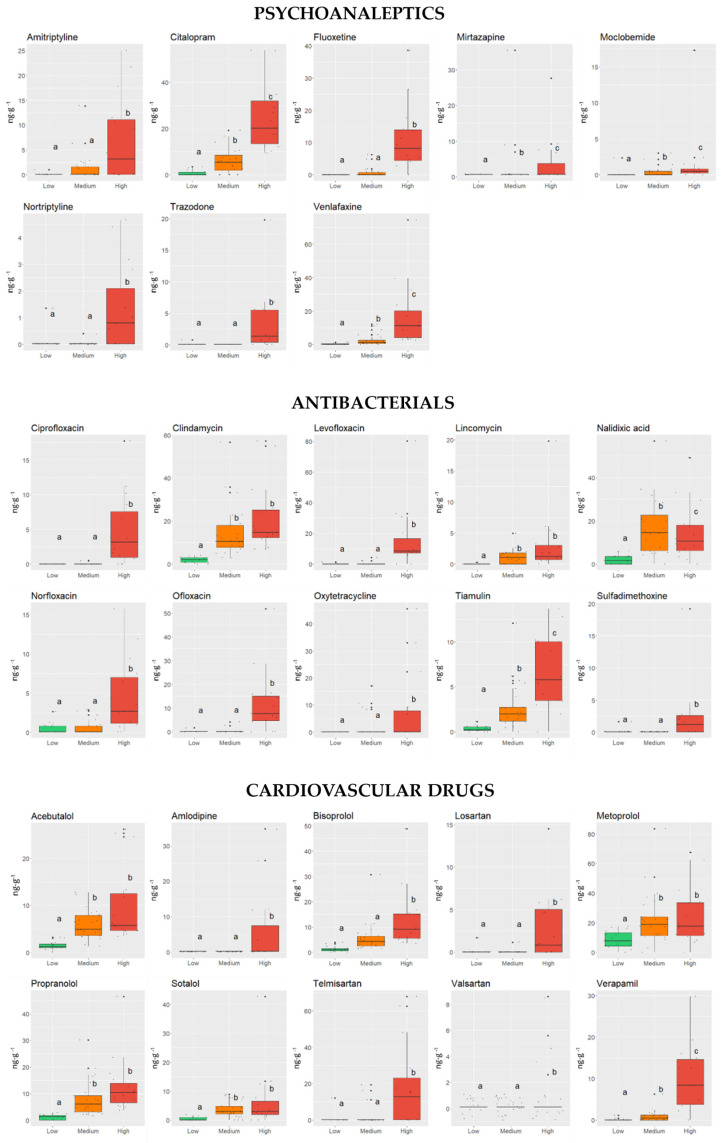

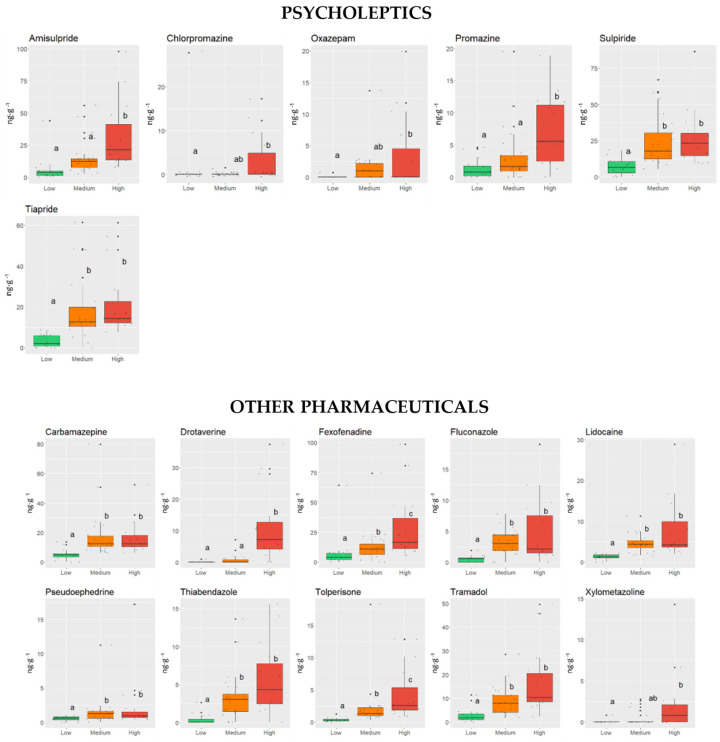
Comparison of PhAC concentrations between different clusters in Figure 3. The statistically non-different groups are denoted with identical letters. The compounds were grouped based on their pharmaceutical action. The central line marks the median, and the lower edge of the box marks the 25th percentile, whereas upper edge marks 75th percentile of the dataset. The whiskers mark the lowest and highest values.

The analysis of variance (ANOVA) was used to compare the concentrations of each PhAC in three sample clusters (Figure 4). It was observed that some compounds differ in their patterns between individual clusters.

The observed differences in the PhAC content among various clusters can be attributed to a range of environmental factors, such as strong river flow, rapid dilution of WWTP effluents, water exchange with the Baltic Sea, salinity variations, mineralogical composition, and TOC content. However, the most likely explanation is closely tied to human activity and various anthropogenic influences. According to Statistics Poland (https://stat.gov.pl/en/, accessed on 21 September 2025), in sparsely populated rural areas around the Szczecin Lagoon, homeowners and businesses rely on septic tanks to dispose wastewater due to lack of access to a municipal sewage system. Leaking septic tanks can contaminate groundwater with PhACs, which, in turn, may reach surface water. The main issue is the illicit discharge of domestic and livestock wastewater directly into agricultural drainage ditches in rural areas, as well as wastewater discharge from ships, leisure boats, and yachts [51,52]. Additional sources of PhAC contamination may include rain, snowmelt, and irrigation runoff from farmland, which is transported through the agricultural drainage system into surface water. In many cases, sewage system malfunctions and WWTP overflows during heavy rain result in untreated sewage being discharged directly into the river. Furthermore, ship movements and dredging activities during maintenance can lead to the resuspension of bottom sediments, contributing to the long-distance transport and dispersion of PhACs from WWTP effluents [55]. Therefore, factors related to the characteristics of the sampling points and their cluster assignments were further explored. For that purpose, analysis of bottom sediment characteristics was conducted to identify environmental and anthropogenic factors influencing the distribution of PhACs in the Odra River estuary. The ANOVA method was employed to examine the relationship between PhAC occurrence and concentration and the physicochemical parameters of the sediments. The following parameters were investigated: pH of pore water, TOC, TN, AVS, TP, As, Ba, Cd, Co, Cr, Cu, Fe, Hg, Ni, Mn, Mo, Pb, Sn, and Zn. Additionally, clay, silt, and sand fractions, as well as distance from WWTP, were included in the analysis.

**Figure 5:**
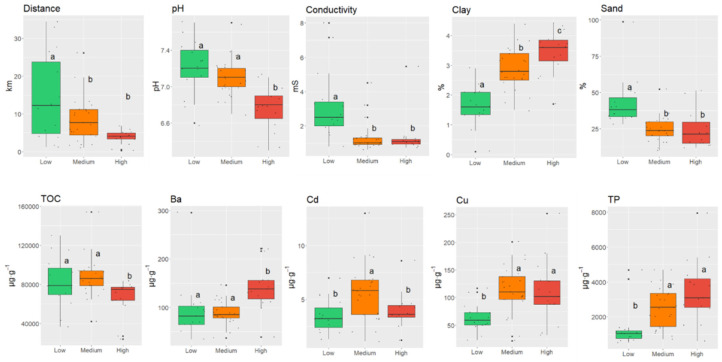
The characteristics of sediment samples that differentiate the three clusters in Figure 3 with low, medium, and high content of PhACs (statistical significance at *p* < 0.05). The central line marks the median, and the lower edge of the box marks the 25th percentile, whereas upper edge marks the 75th percentile of the dataset. The whiskers mark the lowest and highest values. The non-different groups in terms of concentrations of PhAC are denoted with the same letter.

The study by ANOVA revealed that only distance from the WWTP, pH, conductivity, sand and clay fraction, Cu, Cd, and Ba had a significant effect on the cluster assignment of each sampling point (Figure 5).

The Principal Component Analysis (PCA) method was used to reduce and summarize a large number of variables into a smaller number of derived variables that may be readily visualized in 2- or 3-dimensional space. PCA transforms correlated variables into a smaller set of uncorrelated principal components that maintain most of the variance of the original dataset.

**Figure 6:**
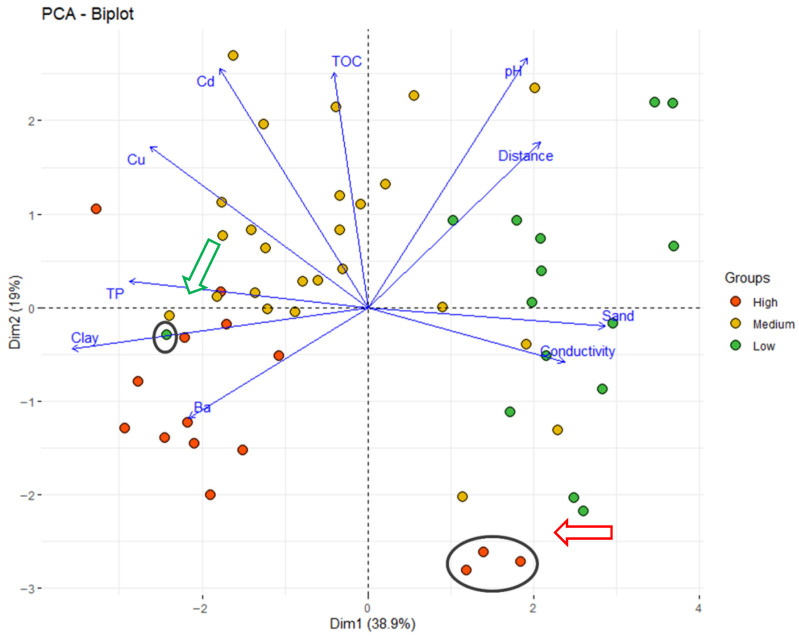
Principal Component Analysis (PCA) of the water/sediment properties in three clusters with high, low, and medium concentrations of PhACs from Figure 3. Arrows indicate sampling points with inadequately low (green arrow) or high (red arrow) concentrations of PhACs regarding sediment properties and distance from the WWTP.

Principal Component Analysis revealed that high and medium PhAC concentrations in sediment samples were associated with elevated TP levels, as well as high clay mineral (Figure 6) and low sand content. TP is formed during the decomposition of water plants or animals; however, elevated concentrations of phosphorous in sediments is likely caused by wastewater discharge. It is worth noting that, in this case, it is not correlated with the distance from the effluent outlets of WWTP. TP may also be washed out from farmland soils. The manure used as a fertilizer contains a lot of various PhACs used in animal husbandry. Overall, additional sources of PhACs in the bottom sediments were likely run-off water from farmland and illicit discharge of wastewater from rural areas. The high PhAC content was associated with the presence of clay minerals in the sediments, which adsorbed both PhACs and metals, particularly Ba. Clay minerals are sourced from post glacial weathered Quaternary till in the bedrock of the Szczecin Lagoon area. The clay minerals are also delivered to the lagoon as suspended solids in the water of the Odra River. The number of negative charge sites on the surface of clay minerals depends on pH. Therefore, a certain relation between pH and a high concentration of PhACs in sediments was observed. High PhAC concentrations correlated not only with increased Ba levels, but also proximity distance to WWTPs. According to a previous study [55], the samples collected close to WWTPs located in the cities of Szczecin, “Pomorzany”, and “Zdroje” were the most contaminated by PhACs. The discharges from those WWTPs was the most significant, among others located in the surrounding area of Szczecin Lagoon (Figure 2). Dispersion of PhACs from wastewater discharge seems to be strongly influenced by the hydrographic conditions and sediment compositions of the Odra River and Szczecin Lagoon; therefore, the concentration of compounds does not show a direct correlation with the distance from the WWTP, nor with the “high”, “medium”, or “low” clusters.

The main source of Ba, which was responsible for the separation of the “high” and “low” clusters, is the weathering of rocks (for example: feldspar, dolomite, limestone). However, in Poland, coal mine drainage water, which is discharged into the Odra River, serves as an additional source of Ba at high concentrations [62]. Inorganic fertilizers based on potassium and calcium also release Ba into surface water. Ba weakly and reversibly adsorbs to clay minerals and it is easily desorbed due to salinity or pH change [63]. Barium may be replaced by PhACs on the surface of clay minerals during the adsorption process. Additionally, similarly to other metal cations, Ba may form insoluble salts with PhACs [64].

The association of low concentrations of PhAC with conductivity may be explained by the hydrographic conditions of the Odra River estuary. Szczecin Lagoon represents a boundary environment between sea and river [65]. Suspended sediments contained mainly fine fractions such as silt, clay, colloidal inorganic (iron oxides, hydroxides), and organic matter (humic and fulvic acids, biofilm) [66,67,68]. Slowing water flow and changing water salinity (conductivity) induced the sedimentation of suspended particles and organic matter that contained adsorbed PhACs. As a result, the bottom sediments in the southern part of the lagoon are rich in organic material whereas sandy sediments prevail in the northern part. The sand fraction does not adsorb organic and inorganic contaminants. As a result, sediment samples collected from the southern part of the lagoon were more contaminated by PhACs than those collected from the northern part. Moreover, in the northern part of the lagoon, there is no significant wastewater discharge from WWTP, which can serve as a source of PhACs. In that area, the treated waste is usually released to drainage ditches or nearby streams that drained water to the Lagoon. However, partial dilution of the contaminants by the occasional inflow of Baltic water is not excluded. It requires further long-term studies to confirm this.

The PCA study also revealed the sampling sites with unexpectedly high or low concentrations of PhACs in the bottom sediments. Those sampling sites are marked in Figure 6. In the sediment sample collected from sampling site # 49, the PhAC content was very low despite the proximity of discharge points from WWTPs in Międzywodzie and Kamień Pomorski, and the mouth of the Świniec River that collected irrigation water from farms. Sampling site # 49 was located in Sokolicki przepływ, a strait connecting Kamieński Lagoon and Wrzosowska Bay. The strait is very narrow (760 m wide), and thus, strong water currents, movements of naval ships, and fishing and leisure boats wash out the PhACs from bottom sediments of the strait. The samples of bottom sediments collected around Ostrów Grabowski, a part of the port of Szczecin, (sampling site # 25, #26, and # 5), were characterized by a high concentration of PhACs and sand fraction. Plausible sources of the PhAC contamination of sampling sites # 25 and #26 were urban allotment gardens, the municipal beach, and the yacht marina located on Grodzka Island. Sampling site #5 was located in Duńczyca, a ship channel, in proximity of the discharge point from a ship wastewater treatment plant. Additionally, there were medical and veterinary waste thermal treatment plant and container terminals on Ostrów Grabowski Island.

Additionally, the relationship between different clay minerals present in bottom sediments and the occurrence of PhACs was also investigated. Smectite (montmorillonite) was prevalent in clay fraction of the most sediment samples (20–50%). Other clay minerals present in the sediment samples were chlorite (3.6–21.3%), illite (2.8–18.9%), and kaolinite (below 5%). In natural environmental conditions, clay minerals are very similar in terms of physicochemical properties [69]. The cation exchange sites are covered by strongly adsorbed metal cations. Thus, the surface of the minerals is neutral and the interaction between clay minerals and PhACs is very weak. The statistical analysis reveals no effect of clay mineral composition on the occurrence and concentration of PhAC in sediments (*p* > > 0.05). However, further research is needed to confirm this, as the variability in the clay mineral composition of the sediment samples used for statistical analysis was very small.

The overall results of the PCA indicated that the occurrence and concentration of PhACs depended mainly on the adsorbing capabilities of the sediment. Strongly adsorbed PhACs can be carried by re-suspended sediment particles to distant locations during estuarine water circulations induced by wind or river surges. Therefore, the effect of water motion in the estuarine of the Odra River on PhAC distribution will be a subject of future investigations.

### 3.2. Influence of Physical and Chemical Parameters of PhACs on Their Occurrence in Sediments

It has been shown that the adsorption of PhAC on bottom sediments depended also on the physicochemical properties of the compounds [10,18,19,20]. Thus, the influence of solubility, Kd (solid/liquid partitioning coefficient), and Kow (octanol/water partitioning coefficient) of PhAC on the occurrence and concentration of the compounds in sediments from the Odra River estuary was investigated. Additionally, yearly consumption of the PhACs in Poland and the percentage of non-metabolized compounds excreted from the human organism were also considered. All those compound properties are presented in Table 1.

**Figure 7:**
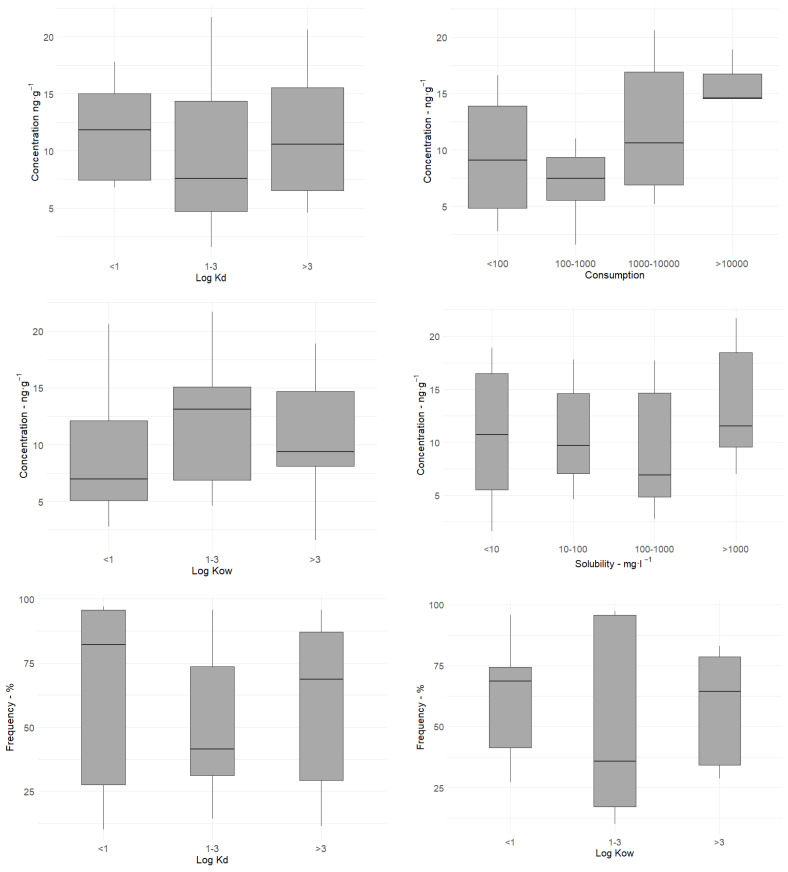

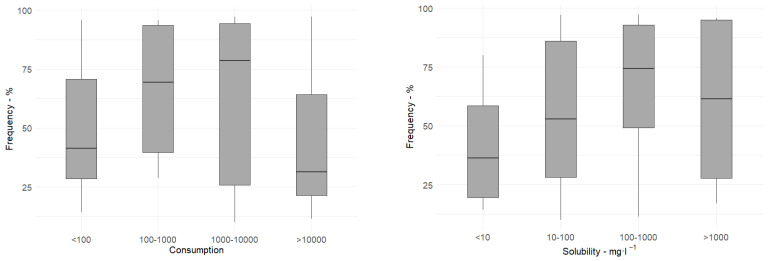
The relation between selected physicochemical parameters and the concentration and detection frequency of PhACs in sediments from the Odra River estuary. The central line marks the median, and the lower edge of the box marks the 25th percentile, whereas the upper edge marks the 75th percentile of the dataset. The whiskers mark the lowest and highest values. Statistically, the groups did not differ significantly from each other (*p* > 0.05).

The ANOVA did not reveal any relationship between the physicochemical properties of the PhACs and their concentration or detection frequency in the sediment samples (Figure 7). However, it was observed that highly lipophilic compounds were more likely to be present at high concentrations in the sediments, while the most soluble compounds were the most widespread.

**Figure 8:**
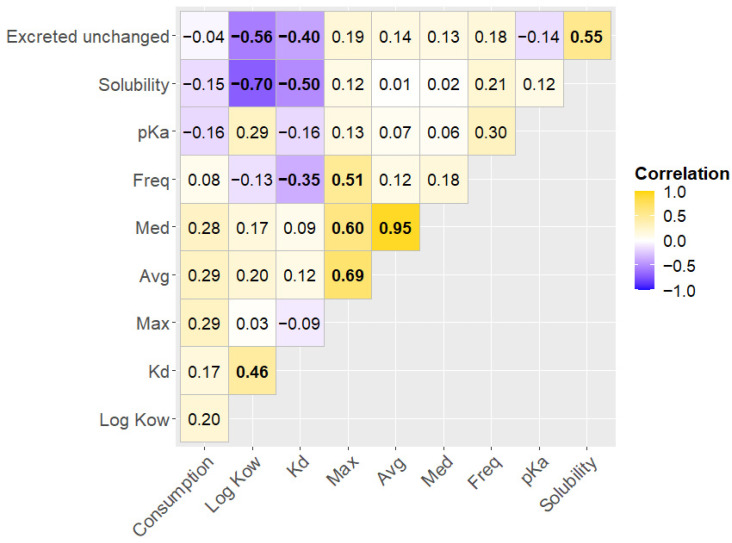
Spearman rank correlation between physicochemical parameters of sediments and the spatial distribution (concentration, detection frequency) of PhACs in the Odra River estuary. Med—median concentration, Avg—average concentration, Max—highest concentration, Freq—detection frequency. Bolded numbers indicate statistically significant correlation coefficients.

Thus, correlation analysis was used to identify the relationship, as there was no need to create value intervals, since continuous variables were analyzed (Figure 8). The results of the analysis revealed a negative relationship between Kd and the detection frequency of compounds in sediment samples. This can be explained by the high mobility of these PhACs in the environment. Compounds with higher Kd are less mobile, binding to sediments closer to the pollution source. Additionally, these compounds are more likely to be resistant to biodegradation and less soluble in water. In general, Kd can be a useful parameter in environmental monitoring, aiding in the identification of the source of pharmaceutical contamination, tracking the movement of PhACs in aquatic environments, and supporting the development of targeted monitoring programs. It was found that the sales data and the percentage of excreted unchanged drugs were not related to the occurrence or concentration of PhACs in the bottom sediments.

**Figure 9:**
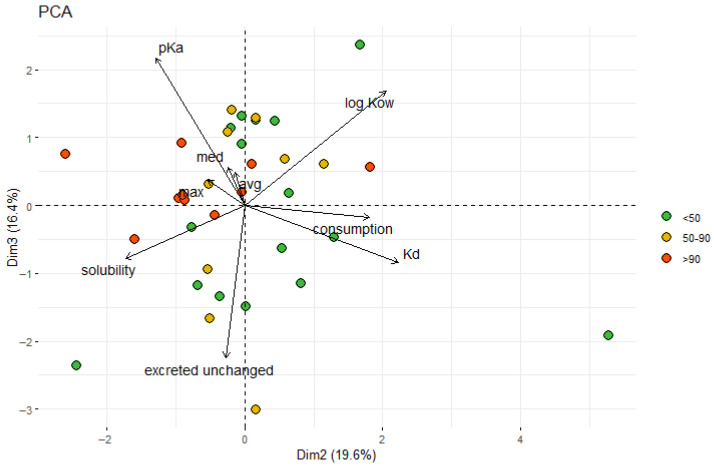
The Principal Component Analysis (PCA) biplot graph shows the properties of PhACs and their detection frequency. The highest frequencies, above 90%, are highlighted in red.

Contrary to correlation analysis, PCA allows us to find interrelations between more than two parameters. The results revealed a relation between the detection frequency of PhACs, solubility in water, and pKa (Figure 9). Those parameters characterized the mobility of the compounds in the environment. More soluble and dissociable compounds are transported with water to the most distant locations where, due to changes in environmental conditions, they sink in bottom sediments. Low-solubility compounds with strong binding affinity to sediment particles are likely deposited close to the contamination source. Thus, the dissociable compounds with high solubility in water are more frequently detected in sediment samples. Additionally, it seems that such compounds are persistent in aquatic environments because they last enough time to be distributed to distant locations. However, further studies are needed to confirm that.

**Figure 10:**
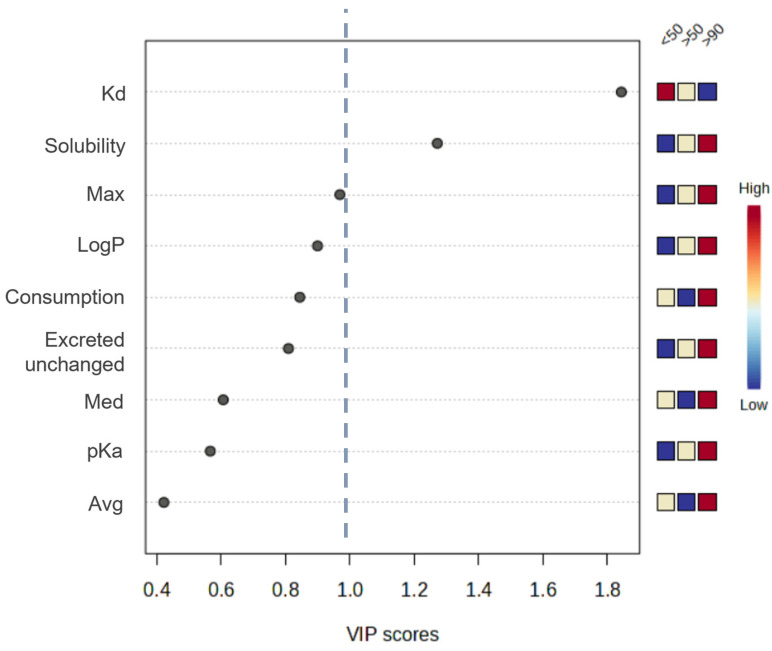
Variable Importance in Projection (VIP) score plot of the PhAC properties that differentiate between PhACs with varying detection frequencies in samples of sediments from the Odra River estuary. The blue line was drawn at a VIP score of 1, the threshold above which a variable is considered significant.

Additionally, Partial Least Squares Discriminant Analysis (PLS-DA) was performed. The VIP (Variable Importance in the Projection) score was used to distinguish factors that significantly affect detection frequency of PhACs in the samples of bottom sediments. The VIP score indicates the statistical significance of each variable in the PLS-DA model, and thus reflects the contribution of the variable to the predictive power of the model. The VIP score is calculated as a weighted sum of the squared correlations between the original variable and the PLS-DA components. The weights are proportional to the percentage of variation explained by each PLS-DA component in the model. The blue line marks a VIP score of 1, and thus, variables with higher scores are considered as statistically significant (Figure 10). It was observed that only two variables—Kd and solubility—exceeded this threshold. This confirms the PCA findings that compounds with low binding affinity to sediments and high water solubility are more frequently detected in bottom sediments.

Overall, the dependence of PhAC distribution on Kd and solubility was suggested in previous review papers [2,26,70]. However, those conclusions were based mainly on a simple comparison of the environmental data available in the literature and physicochemical properties of PhAC and were not supported by multivariate statistical analysis of real datasets.

## 4. Methodology

### 4.1. The Analysis of Clay Minerals in Sediments

The powder X-ray diffraction (PXRD) analyses of clay minerals were conducted at the Faculty of Chemical Technology and Engineering, West Pomeranian University of Technology ZUT, Szczecin Poland. The Empyrean Malvern Panalytical diffractometer (Almelo, The Netherlands), with a Cu radiation source, CuKα = 1.5418 Å, 30 kV/35 mA, spinner 1/16 s, and graphite monochromator, equipped with a PIXcel 3D 1 × 1 detector with a 2θ range from 5 to 80°, was used for the analyses. The samples of ground sediment were dried by lyophilisation. The clayey silt fraction (<0.004 mm) was separated from the sediment samples by a laboratory centrifuge (Thermo Scientific X1 M-20 Rotor, Waltham, MA, USA). Pure-phase ZnO was used as an internal standard: 0.111 g ZnO was added to 1 g of analysed material. The addition of 1 to 3 drops of ethylene glycol was applied for the identification of swelling clays according to Środoń et al. [71]. Rietveld refinement of the XRD pattern was used in the quantification of mineral phases. The RockJock v6 software programme was used for quantitative determination of clays in the sediment samples by comparing the integrated XRD intensities of individual minerals to the intensities of the internal standard according to Eberl [72].

### 4.2. Data Analysis

The data on total organic carbon, (TOC), total nitrogen (TN), total phosphorus (TP), acid volatile sulfide (AVS), As, Ba, Cd, Co, Cr, Cu, Fe, Hg, Mn, Mo, Ni, Pb, Sn, and Zn, as well as 130 pharmaceutically active compounds in bottom sediments, from 70 sampling sites in the Odra River estuary, were examined and published previously [55,56]. All data regarding sampling points, characteristics, and analytical results can be found there. The sampling sites represent different anthropogenic impacts on the environment of the Odra River estuary. The statistical analysis of the results was performed by using R (version 4.3.1, R Foundation for Statistical Computing, Vienna, Austria). Data processing was performed with the Dplyr (1.1.4) package in R. Principal Component Analysis (PCA) was performed for set of variables with the Factoextra (1.0.7) package in R. Box plots were created with the ggplot2 (3.5.1) package in R. The comparison of PhAC concentration means of the three groups (high, medium, low) was performed using the ANOVA/Kruskal–Wallis tests for log10 scaled data showing normal and non-normal distributions, respectively. Levene’s test indicated the equality (homogeneity) of variance for the PhAC concentration variable among these three groups. The Spearman correlation analysis was performed for the set of 10 variables with the rcorr function in Hmisc (5.2-2) in R and correlation was performed with the ggcorrplot (0.1.4.1) package in R. The method of Euclidean distance was used in the Cluster Analysis for the similarity measure, and the method of Ward’s minimum variance was used for the clustering of variables and row dendrograms. PLS-DA analysis was performed using Metaboanalyst 6.0. Consumption of the PhACs was estimated based on the report on refund drugs sold in Poland provided by the Polish Ministry of Health (the data are available on the Internet page of the Ministry).

## 5. Conclusions

The results from previous environmental studies on the physicochemical properties of bottom sediments from the Odra River estuary (SW Baltic Sea) and their contamination by PhACs were compiled and examined using multivariate statistical analysis methods [55,56]. It was found that clay mineral and total phosphorus (TP) content were the major physicochemical properties of the sediments influencing the spatial distribution of PhACs in the bottom sediments, having a greater influence than the distance from the WWTP discharge. However, hydrographic conditions, changing from riverine to marine environments, may also affect the occurrence and concentration of PhACs in the sediments. Sediment distribution coefficients (Kd) and the solubility of PhACs also influenced the occurrence and concentration of the compounds in the estuary. It was found that more soluble PhACs with low adsorption affinity to the sediment were detected more frequently in sediment samples and were transported with water to distant locations, while less soluble compounds with high adsorption affinity sank in bottom sediments close to the contamination source. The results indicated that neither the amount of drug excreted unchanged nor the sales data affected the spatial distribution of the compounds in the bottom sediments. The results of this study are useful in the planning of environmental monitoring. Usually, environmental monitoring includes a long list of potential contaminants [73]. The analysis of those compounds is very laborious and time consuming. Therefore, finding the criteria for prior selection of the most persistent compounds in the environment and reducing the list of monitored substances is very desired by environmental authorities. Additionally, the sampling sites with high concentrations of contaminants may be indicated based on the major sediment parameters governing the spatial distribution of PhAC and linked to their occurrence and concentration. This will reduce the number of samples taken to the analysis and reduce the expenses related to environmental sampling and monitoring.

## Figures and Tables

**Table 1:** Physicochemical properties of pharmaceutically active compounds [55].

PhACs	Sale *, kg	log Kow (calc.)	Kd, L/kg (calc.)	Freq., %	pKa	Solubility, mg/L	Excreted Unchanged, %
Acebutolol	1001.3	1.70	1.8	94.3	13.9	259	40%
Amisulpride	2472.2	1.10	46.8	97.1	9.4	293	60%
Amitriptyline	355.3	4.92	37,045.0	28.6	9.4	10.8	5%
Amlodipine	2508.9	3.00	220.2	10	9.4	75.3	5%
Atropine	1.1	1.80	50.6	17.1	9.4	2200	50%
Bisoprolol	621.0	1.87	2.4	95.7	9.5	71	50%
Carbamazepine	25,964.6	2.45	284.1	97.1	7.0	18	3%
Ciprofloxacin	6873.9	0.28	2.6	27.1	6.1	1350	50%
Citalopram	3.3	3.74	1862.2	70	9.9	31	30%
Clindamycin	6703.74	2.16	4.3	95.7	7.6	30.6	15%
Diclofenac	6439.0	4.51	61.2	80	4.2	2.4	1%
Diltiazem	1499.7	2.80	697.4	25.7	8.9	465	4%
Doxepin	2.3	0.67	8426.3	71.4	9.0	31.6	3%
Fluconazole	691.7	0.50	3873.3	74.3	1.8	1000	80%
Fluoxetine	574.4	4.05	15,223.2	34.3	9.8	14	1%
Levofloxacin	6.6	2.10	3.3	35.7	6.2	54	100%
Lidocaine	52.4	2.44	66.7	95.7	8.0	410	10%
Lincomycin	0.6	0.20	4.3	57.1	7.6	927	33%
Losartan	3788.2	1.19	66,794.0	15.7	5.0	8.2	4%
Metoprolol	409.0	1.88	4.6	92.9	9.7	10,000	5%
Mirtazapine	1.9	2.90	2070.6	14.3	7.9	0	4%
Moclobemide	483.6	10.6	15.2	41.4	10.6	4	1%
Norfloxacin	0.02	−1.03	6.8	41.4	6.3	280	30%
Ofloxacin	46.1	−0.39	3.3	30	6.0	10,000	70%
Oxazepam	0.4	2.24	88.6	27.1	1.7	20	2%
Propranolol	1375.1	3.48	89.4	82.9	9.4	61.7	1%
Sotalol	4155.3	0.24	2.8	68.6	8.2	782	90%
Sulpiride	1829.9	0.57	105.9	95.7	9.1	2280	90%
Telmisartan	12,696.3	7.70	3,319,882.0	31.4	3.6	0	97%
Tiapride	6.9	0.90	44.3	91.4	8.8	252	50%
Tramadol	141.9	1.34	59.0	95.7	9.4	1151	40%
Trazodone	6180.1	2.68	3556.2	22.9	6.7	27.6	1%
Valsartan	16,229.1	1.50	75,161.6	11.4	4.7	180	13%
Venlafaxine	5577.2	3.80	107.5	78.6	10.1	267	5%
Verapamil	462.9	3.79	340,135.6	64.3	8.9	4.5	4%

* Consumption was estimated based on reports on drug reimbursement by the National Health Fund in 2022.

## Data Availability

Data available on request from the authors.
